# Hexacoordinated nitrogen(V) stabilized by high pressure

**DOI:** 10.1038/srep36049

**Published:** 2016-11-03

**Authors:** Dominik Kurzydłowski, Patryk Zaleski-Ejgierd

**Affiliations:** 1 Centre of New Technologies, University of Warsaw, Warsaw 02-097, Poland; 2 Faculty of Mathematics and Natural Sciences, Cardinal Stefan Wyszyński University in Warsaw, Warsaw 01-938, Poland; 3 Institute of Physical Chemistry, Polish Academy of Sciences, Warsaw 01-224, Poland

## Abstract

In all of its known connections nitrogen retains a valence shell electron count of eight therefore satisfying the golden rule of chemistry - the octet rule. Despite the diversity of nitrogen chemistry (with oxidation states ranging from + 5 to −3), and despite numerous efforts, compounds containing nitrogen with a higher electron count (hypervalent nitrogen) remain elusive and are yet to be synthesized. One possible route leading to nitrogen’s hypervalency is the formation of a chemical moiety containing pentavalent nitrogen atoms coordinated by more than four substituents. Here, we present theoretical evidence that a salt containing hexacoordinated nitrogen(V), in the form of an NF_6_^−^ anion, could be synthesized at a modest pressure of 40 GPa (=400 kbar) via spontaneous oxidation of NF_3_ by F_2_. Our results indicate that the synthesis of a new class of compounds containing hypervalent nitrogen is within reach of current high-pressure experimental techniques.

Since the first synthesis of the NF_4_^+^ cation in 1966^1^ numerous experimental attempts have been made to synthesize its neutral parent molecule, NF_5_^2^,^3^,^4^,^5^. All of these attempts turned out fruitless, eventually leading to the conclusion that the highest coordination number (CN) attainable for pentavalent nitrogen is 4, with higher CNs not possible due to steric hindrance around the nitrogen atom^6^. Obviously, it’s possible to find nitrogen in an environment with higher CNs, but only in salts containing the isolated, and non-hypervalent, N^3−^ anion (e.g. Li_3_N exhibiting 8-fold N coordination)^7^, or in certain coordination complexes of trivalent nitrogen (e.g. [(Ph_3_PAu)_5_N]^2+^)^8^ in which, despite the high CNs, the valence shell electron count on nitrogen never exceeds eight.

Theoretical investigations into the properties of the nitrogen pentafluoride molecule (NF_5_) in the gas phase^9^,^10^,^11^,^12^,^13^ indicate that although it *is* a minimum on the potential energy surface (PES)^11^,^12^,^13^, its decomposition into NF_3_ and F_2_ is highly exothermic (+1.82 eV per NF_5_)^12^,^13^. Regarding the geometry of the molecule, the ground state structure of NF_5_ is a trigonal bipyramid with five covalent N–F bonds. Interestingly the energy of formation of the molecule is comparable with that of solid (NF_4_^+^)(F^−^)^14^, making the NF_5_ system an interesting example of the interplay between covalent and ionic bonding.

In this communication we present results of a comprehensive computational investigation on the possibility of synthesizing solid NF_5_ from NF_3_ and F_2_ through the application of external pressure in the range of several dozens GPa (1 GPa = 10 kbar). At present pressures up to 200 GPa are routinely achieved in diamond anvil cells (DACs), and the large influence of such high-pressure (HP) conditions on the properties and reactivity of the elements and chemical compounds is well documented^15^,^16^,^17^,^18^. The potential of obtaining novel species through the application of HP is exemplified inter alia by the recent synthesis of nitrogen analogues of alkanes^19^, or by the theoretical predictions that hypervalent carbon species can be stabilized at large compression^20^.

Most importantly the oxidative strength of fluorine has been predicted to *increase* considerably at elevated pressures with calculations indicating that at such conditions F_2_ should oxidize Cs to CsF_3_^21^,^22^, HgF_2_ to HgF_3_^23^, and Ar to ArF_2_^24^. Furthermore both theoretical^25^,^26^,^27^,^28^ and experimental^19^,^29^,^30^,^31^ high-pressure studies on the N/H system (analogous to the N/F system studied here) indicate that a wealth of exotic N_x_H_y_ structures should, and indeed does stabilize at HP conditions.

## Results

### Computational approach

Our solid-state calculations, performed within the Density Functional Theory, in the 0–300 GPa pressure range, utilized the hybrid HSE06 functional^32^,^33^,^34^. Importantly, benchmark calculations conducted for isolated molecules, indicate that this functional reproduces much better the gas-phase thermodynamic stability of nitrogen fluorides compared to the PBE functional^35^ typically used “by default” in HP solid-state calculations (for more details see sections I and II of the Supplementary Information, SI). Candidate structures of NF_5_ were identified through the application of the USPEX evolutionary algorithm^36^,^37^. The search for the enthalpically best structures revealed a surprising diversity of high-pressure NF_5_ polymorphs exhibiting numerous and versatile bonding patterns. All thermodynamic and structural parameters reported here are obtained with the use of the HSE06 functional.

### Structures of NF_5_

**Figure 1:**
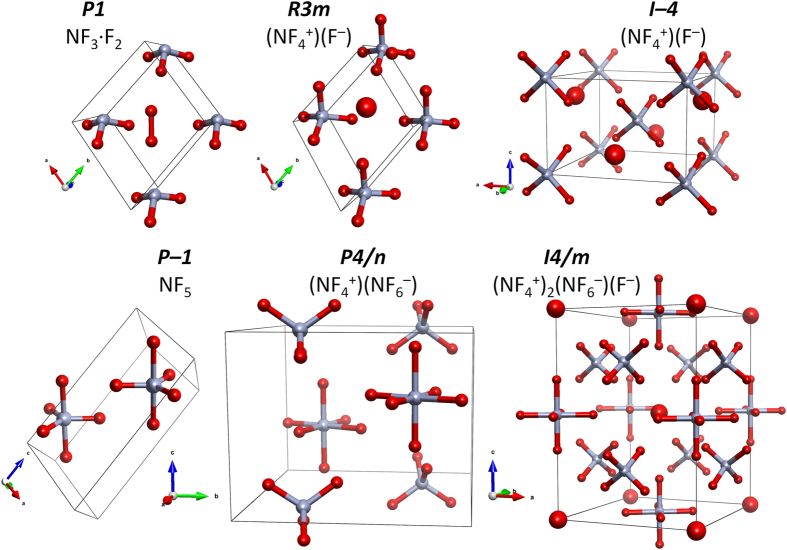
Structures of solid NF_5_. Nitrogen/fluorine atoms are marked by blue/red spheres (covalently bound F atoms are marked with smaller spheres while the F^−^ anions with larger ones).

The lowest enthalpy structures of NF_5_ are shown in Fig. 1; for the purpose of this communication we label them with their respective space group symbols. The *P1* structure is a molecular crystal consisting of NF_3_ and F_2_ molecules, and is the only polymorph containing trivalent nitrogen. Similarly, the *P–1* structure is also a molecular crystal, but in contrast it is composed of *isolated* NF_5_ units. Both *R3m* and *I–4* structures exhibit ionic character and both contain NF_4_^+^ cations and F^−^ anions. The *I4*/*m* and *P4*/*n* phases are also ionic, but apart from the NF_4_^+^ and/or F^−^ ions they both contain the NF_6_^−^ anion in which pentavalent nitrogen is bonded to *six* fluorine atoms. This anion was first proposed by Ewig and Van Wazer^11^ who found it to be dynamically stable in the gas phase, and indeed thermodynamically more stable than NF_5_ + F^−^. To our best knowledge there have been no prior reports on the stabilization of NF_6_^−^ in the solid state.

The assignment of ionic/neutral NF_n_^m+^ fragments is based not only on the fact that the optimized geometry of these fragments agrees well with that predicted by the VSEPR model^38^ (NF_3_ – trigonal pyramid, NF_4_^+^ – tetrahedron, NF_5_ – trigonal bipyramid, NF_6_^−^ – octahedron), but also on the excellent accordance between the bond lengths of these moieties at effectively 0 GPa and those obtained from gas-phase calculations (Table 1). It’s noteworthy to point that even at 300 GPa the NF_n_^m+^ fragments remain well-defined, although in some cases quite distorted (vide infra). This is best exemplified by the fact that at 300 GPa the secondary N···F contacts of all structures are more than 25% longer than the intramolecular N–F bonds, while F···F distances are more than 30% longer compared to the genuine F–F bond in a F_2_ molecular crystal optimized at the same pressure. As expected all of the studied NF_5_ polymorphs are wide-gap insulators with the band gap exceeding 5 eV even at 300 GPa.

The structures optimized with the PBE functional exhibit similar geometries to those described above (obtained with HSE06). Most importantly, we have compared the PBE and HSE06-optimized structure and found no evidence of a Peierls distortion ensuing after optimization of the PBE structures conducted with HSE06, in contrast to what was found for a polymeric phase of nitrogen^39^. We attribute it to the fact that the studied structures contain isolated ions or molecules, and do not exhibit extended motifs (chains, layers) for which one can expect a Peierls distortion. Finally, we note that while the resulting geometries remain essentially identical for the two functionals the relative enthalpies of various structures differ quite substantially; as already mentioned, in this report we focus on the HSE06 values (for details on the PBE/HSE06 enthalpy differences see Section II of SI).

### Stability and pressure evolution of NF_5_ polymorphs

**Figure 2:**
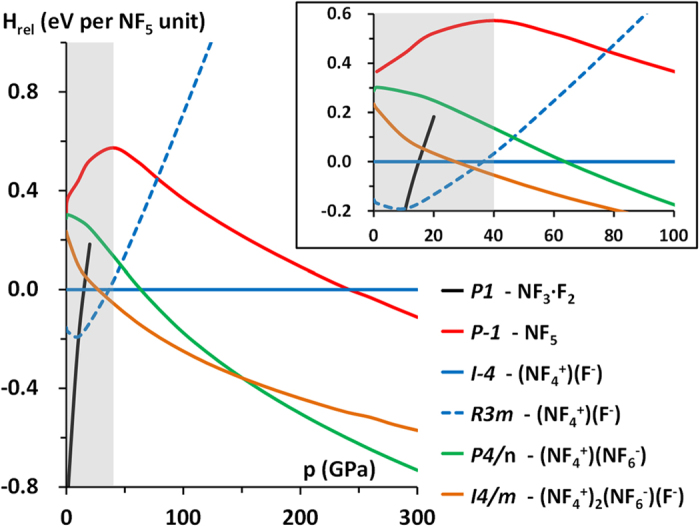
Pressure dependence of the relative enthalpy of NF_5_ polymorphs. At each pressure point the enthalpies (obtained with HSE06 calculations) are referenced to that of *I–4*. The grey region marks the pressure range where the enthalpy change of the reaction NF_3(s)_ + F_2(s)_ → NF_5(s)_ is positive (p < 40 GPa) indicating instability of NF_5_ towards decomposition into NF_3_ and F_2_.

Surprisingly, despite the large diversity of bonding patterns exhibited by the structures containing pentavalent nitrogen their relative enthalpies at low pressure fall in a narrow range of 0.6 eV per NF_5_ unit (≈58 kJ/mol); see Fig. 2.

At pressures lower than 11 GPa it is the *P1* phase (*Z* = 1), containing molecular N^III^F_3_ mixed with F_2_, which is the lowest enthalpy structure. Interestingly upon compression of the *P1* structure the F–F bond in F_2_ lengthens from 1.39 Å at 0 GPa to 1.44 Å at 39 GPa, while the shortest secondary N···F contact contracts from 3.14 Å to 1.89 Å. This points to a significant pressure-induced enhancement of the donor-acceptor interactions between the HOMO of NF_3_ and the antibonding LUMO of F_2_. In fact, upon compression to 40 GPa this interaction leads to heterolytic dissociation of the F_2_ molecules and formation of an ionic structure containing NF_4_^+^ cations separated by F^−^ anions. At 40 GPa the shortest F···F contact in *P1* is 2.17 Å and the four N–F bonds have a length of 1.29 Å. Those changes clearly illustrate that compression of the *P1* phase up to 40 GPa leads to spontaneous oxidation of N^III^F_3_ by F_2_ and subsequent formation of N^V^F_4_^+^ and F^−^.

Note that at 40 GPa the oxidized *P1* phase turns out to be identical in terms of geometry with another NF_5_ polymorph, *R3m (Z* = 3, Fig. 1). The coordination of the F^−^ anion in the latter structure is such that each F^−^ is surrounded by 11 F atoms originating from 7 NF_4_^+^ cations. In particular, the *R3m* polymorph becomes the ground state structure of the NF_5_ system already above 11 GPa. Our USPEX searches identify yet another (NF_4_^+^)(F^−^) structure of the *I–4* symmetry (*Z* = 2), which is even more densely packed than the *R3m* (F^−^ anions surrounded by 12 F atoms originating from 8 NF_4_^+^ cations). This structure is noteworthy since the *I–4* phase becomes more stable than the *R3m* polymorph at 37 GPa. Nevertheless, already above 33 GPa both *I–4* and *R3m* have a higher enthalpy than a more complex *I4*/*m* phase (*Z* = 6), which remains the lowest-enthalpy structure up to 151 GPa (see Fig. 2).

The *I4*/*m* phase is characterized by alternating (NF_6_^−^)(F^−^) and (NF_4_^+^)_2_ layers leading to a general formula of (NF_4_^+^)_2_(NF_6_^−^)(F^−^) = 3NF_5_. This structure bears many similarities to the HP phase of PCl_5_ (*I2*/*m* symmetry) which is best formulated as (PCl_4_^+^)_2_(PCl_6_^−^)(Cl^−^)^40^. The *I2*/*m* polymorph is also layered, but in contrast to the *I4*/*m* structure of NF_5_ it exhibits tilting of the complex ions about an axis lying in the plane of the layers. Geometry optimization of a NF_5_ polymorph isostructural with *I2*/*m* indicated that such a structure is not competitive with *I4*/*m* in terms of enthalpy.

The NF_6_^−^ anion in *I4*/*m* exhibits a slight tetragonal distortion at the low pressure limit (effectively 0 GPa) with two axial N–F bonds shorter by 1.3% than the four equatorial ones. Still all the bond lengths are very close to those obtained in molecular calculation (Table 1). The difference between the axial and equatorial bonds in *I4*/*m* remains nearly constant with increasing pressure and does not exceed 1% at 300 GPa. Upon compression of *I4*/*m* the N–F bonds in NF_4_^+^ shorten by 5% (to 1.25 Å at 300 GPa), while in case of the axial/equatorial bonds of NF_6_^−^ the contraction is approximately 8%.

**Figure 3:**
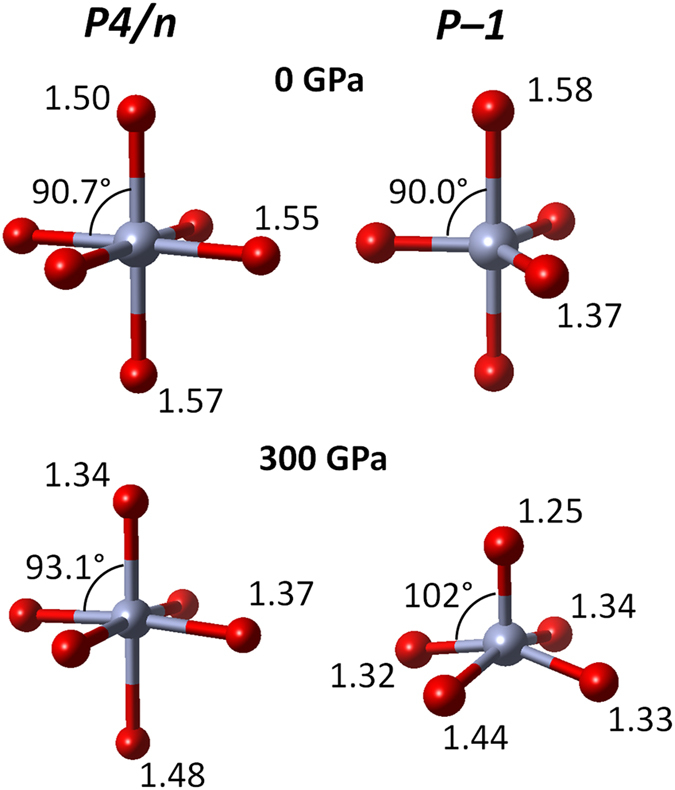
The geometry of the NF_6_^−^ and NF_5_ fragments. The NF_6_^−^ fragment in *P4/n* at 0 and 300 GPa (left) and the NF_5_ fragment in *P–1* at the same pressures (right). Bond distances (in Å) and angles between the axial and equatorial bonds are indicated.

Above 151 GPa *I4*/*m* is predicted to become thermodynamically less stable than a *P4*/*n* structure (*Z* = 4) containing a 1:1 ratio of NF_4_^+^ and NF_6_^−^, and no F^−^ anions. This polymorph is isostructural with the ambient pressure phase of PCl_5_ = (PCl_4_^+^)(PCl_6_^−^)^41^. In *P4*/*n* the NF_6_^−^ anions are more distorted compared to *I4*/*m* with two unequal axial N–F bonds (Table 1 and Fig. 3). High pressure enhances this distortion with the difference between the two axial bonds reaching 10% at pressures above 20 GPa. This seems to indicate that in *P4*/*n* the NF_6_^−^ anion could be also described as a complex of square pyramidal NF_5_ with an F^−^ anion (see Fig. 3). Unequivocal determination which of the two alternative descriptions (highly distorted NF_6_^−^ vs NF_5_···F^−^ complex) is correct requires more elaborate calculations which are beyond the scope of this communication.

Interestingly there is no pressure region in which a structure containing NF_5_ molecules would be the most stable polymorph of NF_5_. The *P–1* structure, which contains such units, becomes more stable than *R3m*/*I–4* at 78/243 GPa, but still in the whole pressure region studied it remains less stable than the NF_6_-containing polymorphs (*I4*/*m* and *P4*/*n*). The coordination of NF_5_ molecules in the *P1* phase changes from trigonal bipyramidal to square pyramidal above 40 GPa (Fig. 3). This transformation, which is in agreement with the predicted non-rigidness of the NF_5_ molecule^12^, is accompanied by a reduction in volume, and a change in the slope of the relative enthalpy (Fig. 2).

## Discussion

The enthalpy change associated with the reaction: NF_3_ + F_2_ → NF_5_ becomes negative already at 40 GPa, as indicated by the grey region in Fig. 2. This shows that NF_5_, containing hypervalent nitrogen, could be synthesized from NF_3_ and F_2_ already at relatively low pressure, in the form of a novel salt (NF_4_^+^)_2_(NF_6_^−^)(F^−^) (*I4*/*m* polymorph). Furthermore, we found NF_5_ to be stable against decomposition into NF_4_, another possible fluorine-rich phase of the N/F phase diagram (see Section III of the SI). Also, we emphasize that the calculated phonons remain positive within the whole Brillouin zone; a proof that the *I4*/*m* phase is dynamically stable both at the pressure of synthesis (40 GPa) as well as at higher pressures (see section IV in SI).

Our results hint that the high-pressure oxidation of NF_3_ by F_2_ is accompanied by the ionization of the reactants, that is formation of NF_4_^+^, NF_6_^−^ and F^−^ ions in place of neutral NF_5_ molecules. The tendency for heterolytic, rather than homolytic splitting of the F–F bond in F_2_ reacting with NF_3_ is further exemplified by the molecular-ionic transition observed in the *P1* polymorph of NF_5_.

On a side note, we here point that for the related N/H system Qian *et al*. also predict formation of ionic species at high pressure and at large hydrogen contents^28^. However these phases contain solely *normal* valent species (NH_4_^+^, H^+^, NH_2_^−^, H^−^), in contrast to the hypervalent NF_5_, NF_6_^−^ species reported in this study. Another difference is that for the N/H system the NH_5_ composition is metastable with respect to decomposition into stable NH_4_ and H_2_ while we find NF_4_ to be unstable at both ambient and high pressure. The instability of hypervalent N/H molecules can be traced back to the large steric crowding around the nitrogen atom in NH_n_ (n > 4) species. In fact, as calculated by Ewig and van Wazer, the NH_5_ molecule is not only thermodynamically but also dynamically unstable in the gas phase, in contrast to NF_5_ which is dynamically stable^10^.

The propensity for the formation of ionic phases at HP, observed also for other nitrogen compounds (NH_3_, N_2_O)^25^,^29^,^42^, can be explained by the pressure-induced increase of the lattice enthalpy (H_L_) of ionic phases which leads to additional stabilization of such structures with respect to molecular, van der Waals bonded polymorphs. The increase in H_L_ is a consequence of the volume reduction upon compression, as the lattice enthalpy is proportional to the inverse cube root of the molecular volume (the so-called Bartlett’s relationship)^43^,^44^.

Interestingly in the case of the NF_5_ system the stabilization of ionic phases leads to larger than expected increase in hypervalency – the NF_6_^−^ ion with a valence electron count of 12 is more stable at HP than the NF_5_ containing 10 electrons in the nitrogen valence shell. We note that this is not a general trend; in the case of hypervalent XeF_2_ the predicted pressure-induced ionization stabilizes a non-hypervalent salt of the (XeF^+^)(F^−^) stoichiometry^45^.

In summary our calculations indicate that a compound containing nitrogen(V) covalently bound by six fluorine atoms can be synthesized via a HP reaction between NF_3_ and F_2_. A newly formed salt, of (NF_4_^+^)_2_(NF_6_^−^)(F^−^) stoichiometry, would constitute the first example of a compound containing hypervalent nitrogen atoms. Most interestingly, due to relatively low pressures involved and due to the involvement of strong ionic interactions this new species might be stable even upon decompression to ambient pressure, particularly at low temperatures. Due to the computer-intensive nature of phonon calculations a detailed study of the dynamic stability of various NF_5_ phases at low pressure is beyond the scope of this study.

Finally, it is also worth to remark that the phase transitions of NF_5_ bear many similarities to those exhibited by PCl_5_, which serves as good example on the rule of thumb that at HP elements tend to resemble their heavier congeners^15^. We hope that the results presented here, which offer an intriguing extension of the palette of N/F binary compounds^46^, will motivate experiments aimed at stabilizing the first genuine hexacoordinated binary compound of pentavalent nitrogen.

## Methods

### Hybrid potential calculations

Periodic DFT calculations utilized the HSE06 hybrid potential^32^,^33^,^34^, while the PBE exchange correlation functional^35^ was used in evolutionary searches, phonon calculations, and for comparative calculations. The projector augmented-wave (PAW) method was used, as implemented in the VASP 5.2 code^47^,^48^,^49^. The cut-off energy of the plane waves was set to 1000 eV with a self-consistent-field convergence criterion of 10^−6^ eV. Valence electrons were treated explicitly, while standard VASP pseudopotentials were used for the description of core electrons. The *k*-point mesh was set at *2π* × 0.06 Å^−1^. All structures were optimized using a conjugate gradient algorithm until the forces acting on the atoms were smaller than 10 meV/Å. The abovementioned parameters ensured convergence of the calculated enthalpy within 2 meV per atom.

### Structure searches

The candidate structures of NF_3_, F_2_, NF_5_, as well as NF_4_ (see SI) were identified with the use of the USPEX evolutionary algorithm coupled with the PBE functional. Evolutionary searches were conducted for *Z* = 1, 2, 3, and 4 at *P* = 50, 100, 200 and 300 GPa. Due to the large computational cost of the HSE06 functional we were not able to employ it during the USPEX runs. Therefore, all of the best candidate structures obtained with USPEX were fully re-optimized (*i.e.* optimization of lattice parameters and internal coordinates) using the HSE06 functional. Beside the best structures identified at PBE level, we also used a number of enthalpically low lying meta-stable structures in the HSE06 re-optimization. For NF_3_ our structure search identified the *Pnma*, *Pnma (2)*, and *P2*_*1*_*2*_*1*_*2*_*1*_ molecular phases proposed in an earlier study^50^, and did not find any new phases which would be competitive in terms of enthalpy with those three. The enthalpy change of the reaction NF_3(s)_  + F_2(s)_ → NF_5(s)_ was calculated taking (at each pressure) the lowest enthalpy polymorph of NF_5_ and NF_3_, as well as the ambient-pressure α polymorph of F_2_^51^. We note that above 50 GPa α-F_2_ (*C2*/*c* space group) symmetrizes spontaneously to a *Cmca* structure which is analogous to the high-pressure polymorph of Cl_2_^52^. Even at 300 GPa F_2_ remains in the form of a molecular crystal.

### Dispersion corrections

In order to determine the influence of dispersion-type interactions on the relative stability of NF_5_ polymorphs we have calculated dispersion corrections (in the form of D3 correction proposed by Grimme and co-workers^53^,^54^,^55^) for structures optimized at the HSE06 level of theory. We found that the D3 correction has very little to no influence on the relative stability of different NF_5_ polymorphs (changes of transition pressures do not exceed 2 GPa upon inclusion of D3 corrections).

Structure visualization was performed with the VESTA 3.1 software^56^. Symmetry recognition was performed with the online program FINDSYM^57^.

## Additional Information

**How to cite this article**: Kurzydłowski, D. and Zaleski-Ejgierd, P. Hexacoordinated nitrogen(V) stabilized by high pressure. *Sci. Rep.* **6**, 36049; doi: 10.1038/srep36049 (2016).

**Publisher’s note**: Springer Nature remains neutral with regard to jurisdictional claims in published maps and institutional affiliations.

## Supplementary Material

Supplementary Information

## Figures and Tables

**Table 1:** Comparison of calculated N–F bond lengths (in Å) of NF_n_^m+^ moieties in the gas phase and in various NF_5_ phases (ax – axial, eq – equatorial bonds).

Moiety	NF_5_ polymorph	Gas phase^a^	0 GPa
NF_3_	*P1*	1.36 (x3)	1.37 (x3)
(NF_4_^+^)	*R3m*	1.30 (x4)	1.31 (x4)
	*I*–*4*		1.31 (x4)
	*P4*/*n*		1.31 (x4)
	*I4*/*m*		1.31 (x4)
NF_5_	*P*–*1*	ax: 1.58 (x2)	ax: 1.58 (x2)
		eq: 1.36 (x3)	eq: 1.37 (x3)
NF_6_^−^	*I4*/*m*	1.55 (x6)	ax: 1.53 (x2)
			eq: 1.55 (x4)
	*P4*/*n*		ax: 1.50, 1.57
			eq: 1.55 (x4)

^a^Values obtained by geometry optimization of molecular fragments utilizing the HSE06 functional and the *cc-pVQZ* basis set.
