# Supplementary material for: Hexacoordinated nitrogen(V) stabilized by high pressure
Source: Sci Rep. 2016 Nov 3;6:36049. doi: 10.1038/srep36049 (PMC5093683; doi:10.1038/srep36049)
Supplement: Supplementary Information [file srep36049-s1.pdf]

SUPPORTING INFORMATION

Hexacoordinated nitrogen(V) stabilized by high pressure

Dominik Kurzydłowski, Patryk Zaleski-Ejgierd

Table of contents

I. Benchmark calculations 2

II. Comparison between enthalpy/pressure curves obtained with PBE and HSE06 3

III. Stability of NF_5 against decomposition into NF_4 5

IV. Phonon stability of *I4/m* and *P4/n* 7

V. Structures of NF_5 at 20 GPa in VASP format 9

VI. References 12

I. Benchmark calculations

Table S1 gives the ZPE corrected atomization energies (in eV) of NF_n^{m+} molecules obtained with the use of the PBE and HSE06 functionals, compared to previously reported CCSD(T) calculations.[1] Our calculations were made using Gaussian09[2] with the *cc-pVQZ* basis set. For both PBE and HSE06 full geometry optimization was conducted.

Table S1: The ZPE corrected atomization energies (in eV).

	PBE	HSE06	CCSD(T)*	Exp.
**NF_3**	–3.19	–1.47	–1.22	–1.37**
**NF_4^+**	6.77	8.88	9.39	–
**NF_5**	–2.75	0.19	0.54	–
**NF_6^-**	–6.84	–3.16	–	–

* Data taken from Ref. 1. To facilitate comparison with our PBE/HSE06 results we have taken the results of *cc-pVQZ* calculations not extrapolated to the complete basis set (CBS) limit; ** data from Ref. 3.

As illustrated PBE-derived atomization energies are more negative than the CCSD(T) results by 1.97, 2.62 and 3.29 eV for NF_3, NF_4^+ and NF_5 respectively, while the values obtained with HSE06 show much smaller discrepancy – they are more negative by 0.25, 0.51, and 0.35 eV for the same set of molecules. This indicates that the PBE functional severely overbinds NF_n^{m+} molecules, even in the case of the non-hypervalent NF_3.

II. Comparison between enthalpy/pressure curves obtained with PBE and HSE06

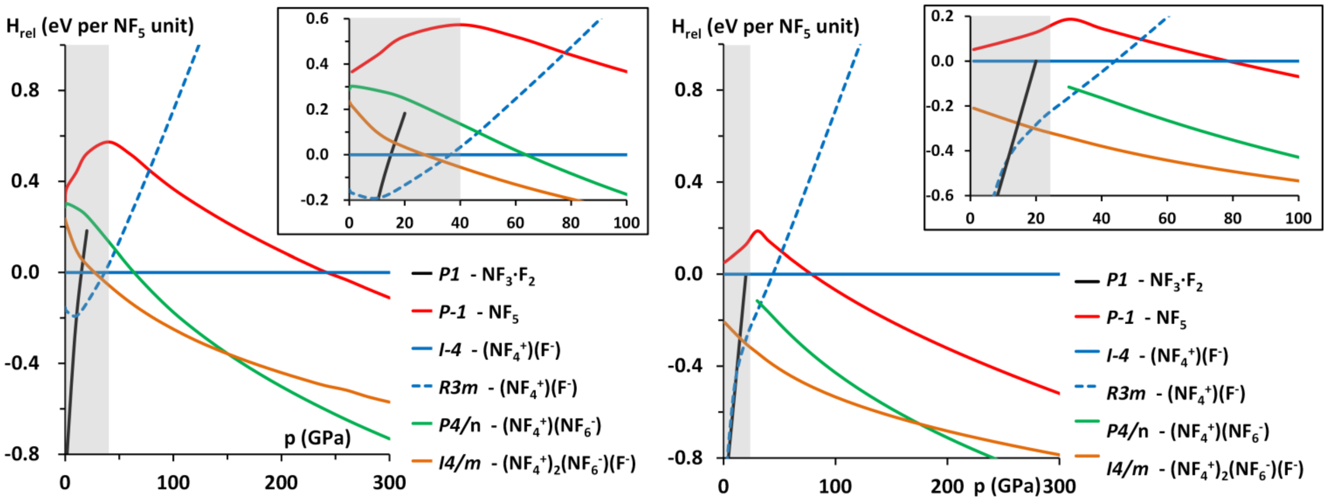

Fig. S 1 Comparison between the pressure dependence of the relative enthalpy of NF_5 polymorphs (referenced to that of *I–4*) obtained with HSE06 (left) and PBE (right) calculations. In both cases the grey region marks the pressure range in which the enthalpy change of the reaction $NF_3 + F_2 \rightarrow NF_5$ is positive (P < 40 GPa for HSE06; P < 23 GPa for PBE). Note that for PBE calculations the *R3m* polymorph transforms to a phase containing molecular NF_3 and F_2 at pressure below 30 GPa, while it retains its ionic structure in the case of HSE06 calculations.

The main difference between HSE06 and PBE results (Fig. S 1) is that at the PBE level of theory polymorphs containing hyper-coordinated nitrogen(V), in the form of either NF_5 (*P–1*) or NF_6^- (*P4/n* and *I4/m)*, are stabilized by 0.2 – 0.3 eV per NF_5 unit with respect to structures containing non-hypervalent nitrogen (*P1*, *I–4*, *R3m*). This has a profound influence on the transition pressures with the *P–1* to *I–4* transition pressure shifting more than 160 GPa from the HSE06 value of 243 GPa to 52 GPa at the PBE level. Similarly, the *P–1* to *I–4* transition pressure shifts from 78 GPa to 52 GPa. The same difference can be seen when comparing pressures at which the *R3m* phase, of $(NF_4^+)(F^-)$ composition, transforms to *I4/m*, of $(NF_4^+)_2(NF_6^-)(F^-)$ composition: the HSE06 value for this phase transition is 48 GPa compared to a much smaller value of 19 GPa at the PBE level of theory.

We note that in the case of transitions between polymorphs containing non-hypervalent nitrogen the pressure shifts are much smaller, as can be seen in Table S3. Finally, it must be noted that at the PBE level of theory the enthalpy change associated with the reaction $NF_3 + F_2 \rightarrow NF_5$ (*I4/m* polymorph) becomes negative already at 23 GPa compared to a value of 40 GPa for HSE06. Therefore, the pressure at which NF_5 might be synthesized is underestimated by a factor of about 2 when comparing PBE and HSE06 results.

Table S2: Comparison of the transition pressures (in GPa) between various NF_5 polymorphs. The PBE values that differ from HSE06 ones more than 10 GPa are highlighted in orange. Structures containing hypervalent nitrogen are underlined.

**HSE06**	*P1*	*P–1*	*I–4*	*R3m*	*P4/n*	*I4/m*	**PBE**	*P1*	*P–1*	*I–4*	*R3m*	*P4/n*	*I4/m*
P1		–	16	11	–	–	*P1*		–	21	12	–	–
P–1			243	78	–	–	*P–1*			80	52	–	–
I–4				37	64	27	*I–4*				44	–	–
R3m					48	33	*R3m*					33	19
P4/n						151	*P4/n*						178
I4/m							*I4/m*						

III. Stability of NF_5 against decomposition into NF_4

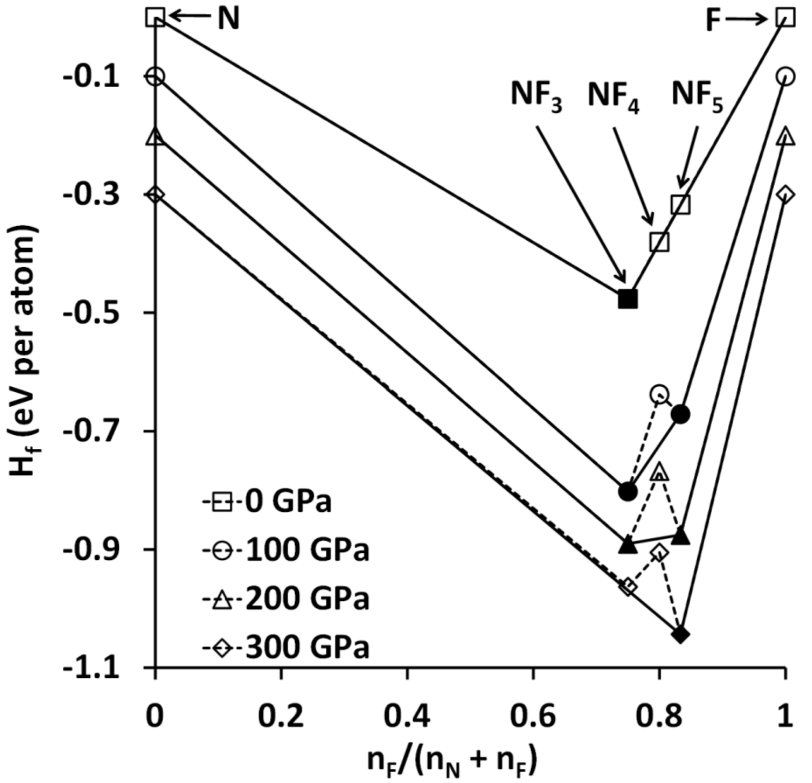

Fig. S 2 Convex hull of the N/F system at 0, 100, 200, 300 GPa calculated with the HSE06 functional. The full and opened symbols indicate stable and metastable compositions, respectively. For clarity the convex hulls at 100, 200, and 300 GPa are shifted by –0.1, –0.2, –0.3 eV, respectively.

In order to assess the stability of NF_5 against decomposition into NF_4 we have calculated the convex hull of the N/F system at 0, 100, 200, and 300 GPa. To find the best structures of NF_4 at a given pressure we have employed the evolutionary searches as described in the main text of the article, and re-optimized the low-enthalpy structures with the HSE06 functional.

As can be seen from Fig. S 2 the NF_4 composition is metastable in the whole pressure range studied. It is close to being stable only at 0 GPa. At these conditions the most stable crystal structure of NF_4 is composed of a mixture of NF_3 and F_2 molecules in a 2:1 ratio (Fig. S 3). At higher compressions the most stable polymorphs of NF_4 consist of either NF_5 and NF_3 molecules (100 GPa) or exotic N_2F_8 molecules (200 and 300 GPa) – see Fig. S 3 below. The lack of stability of NF_4, in contrast to NF_5, can be attributed to the fact that in the former composition an ionic structure containing the NF_4^+ and NF_6^-/F^- ions cannot be realised.

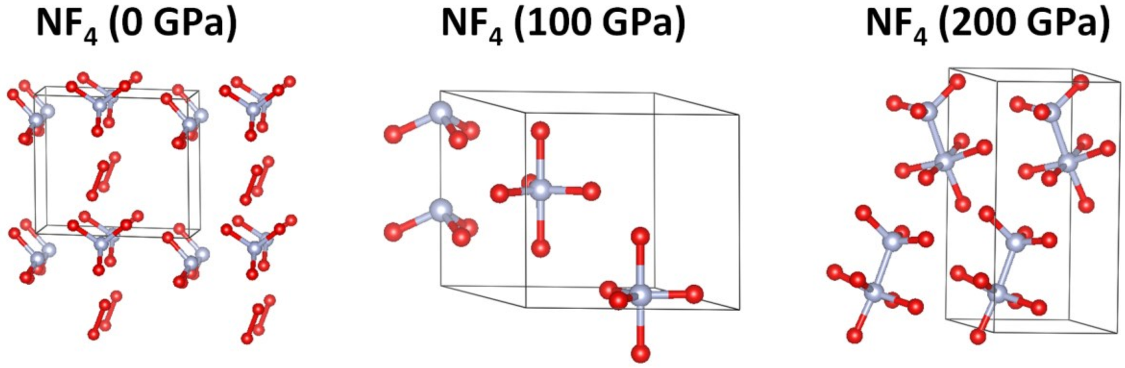

Fig. S 3 Most stable structures of NF_4 at 0, 100, and 200 GPa.

IV. Phonon stability of *I4/m* and *P4/n*

Calculations of the phonon density of state and dispersion curves were conducted with the use of the CASTEP code[4] implemented in the Materials Studio package. These calculations utilized the PBE functional and ultrasoft pseudopotentials, and were carried out on structure optimized with the same functional (PBE). The *k*-point spacing was set at 2π x 0.01 $Å^{-1}$, the cut-off energy of the plane waves was equal to 330 eV with a self-consistent-field convergence of 10^{-6} eV per atom. Phonon calculations were conducted with the finite-displacement method for pre-optimized structures. Supercells of 2x2x2 size were used; they contained 120 and 160 atoms, respectively for *I4/m* at 40 GPa and *P4/n* at 150 GPa.

Below we show the phonon DOS and dispersion curves for *I4/m* optimized at 40 GPa, and *P4/n* optimized at 150 GPa. As can be seen no imaginary modes are observed at these pressures. We also do not find any imaginary modes at higher pressures (P > 40 for *I4/m* and P > 150 for *P4/n*).

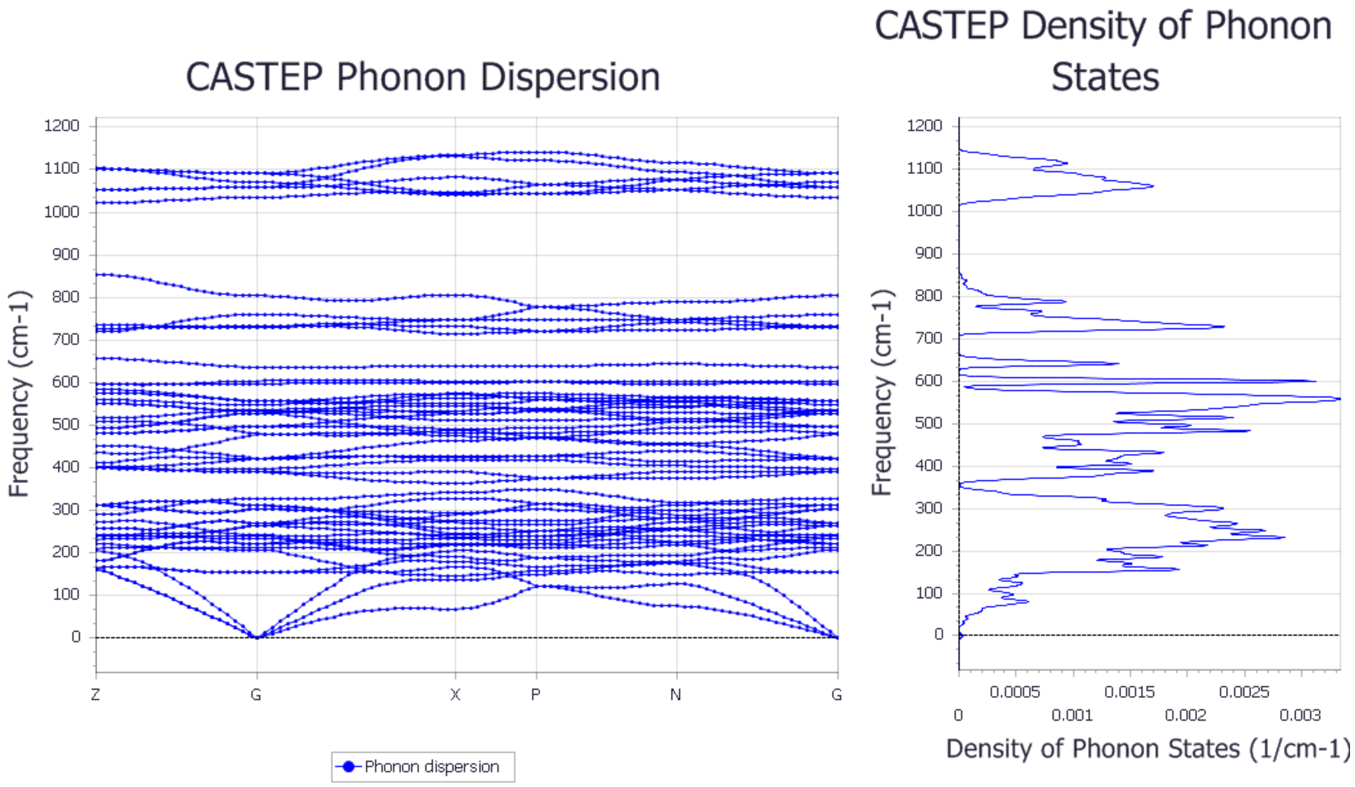

Fig. S 4 Phonon dispersions and the phonon density of states of the *I4/m* structure at 40 GPa. The small imaginary frequency at Γ-point is a numerical artifact.

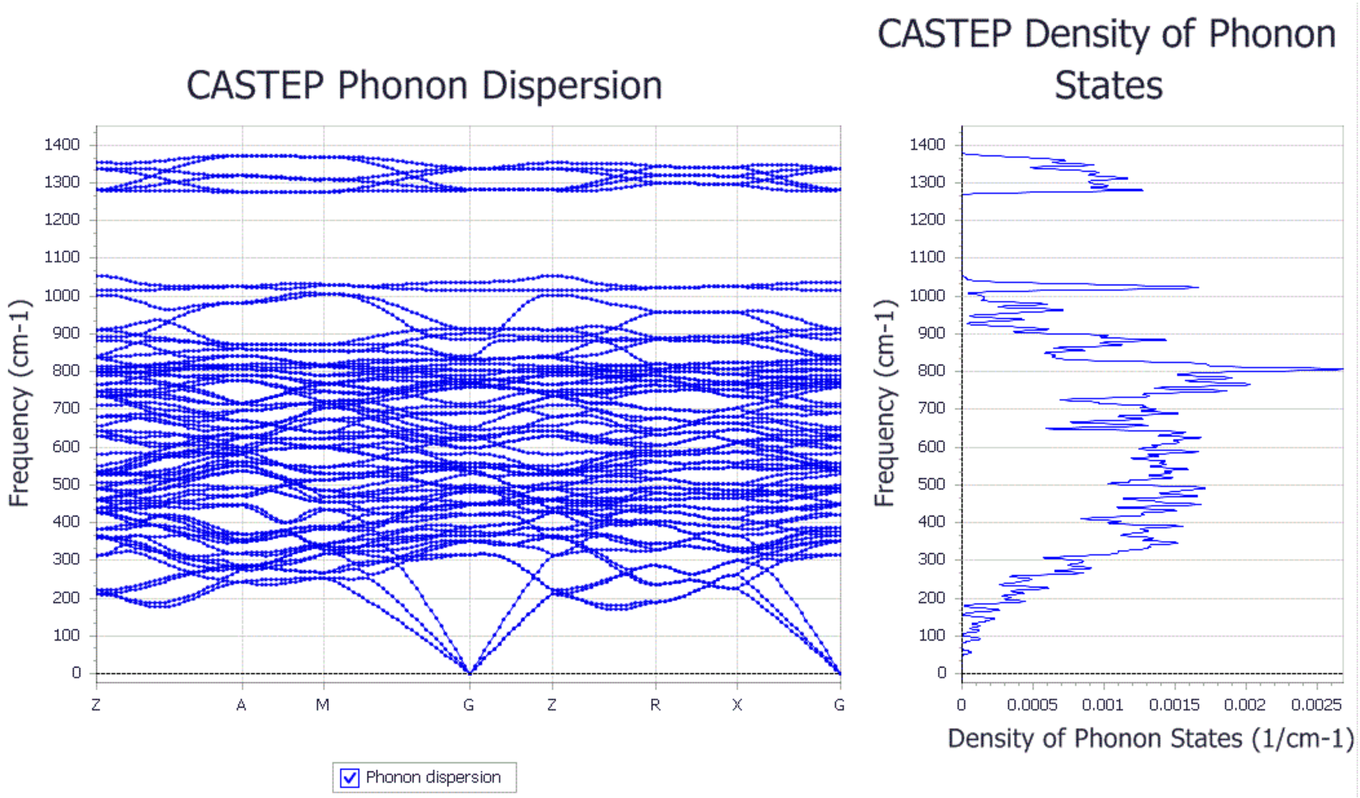

Fig. S 5 Phonon dispersions and the phonon density of states of the *P4/n* structure at 150 GPa.

V. Structures of NF_5 at 20 GPa in VASP format

P1

```
P1
 1.00000000000000000
    4.0150154640006575    0.1105610760940731    0.0122471911669889
    1.1723220094574669    3.8589152773545692    0.0127409887956812
    1.3550870544216098    1.0280784106047529    3.6240959214003419
  N    F
    1     5
Direct
  0.9762095406680515  0.9656095271665137  0.8720102590060677
  0.3283358055673418  0.3171025732405823  0.6313741286071277
  0.7011361065011172  0.1373081778677943  0.1060103674220230
  0.8060693018717657  0.7942200495761682  0.7709758920346417
  0.1499528786900441  0.6930487882955134  0.1052367325977102
  0.5676764057016825  0.5529908998534196  0.4937526643324333
```

R3m

```
R3m
1.0
        4.5033597946         0.0000000000         0.0000000000
       -2.2516798973         3.9000239845         0.0000000000
        0.0000000000         0.0000000000         8.7467498779
   N    F
   3   15
Direct
     0.000000000         0.000000000         0.891910017
     0.666666687         0.333333343         0.225243345
     0.333333343         0.666666687         0.558576703
     0.822380006         0.177619994         0.279159993
     0.822380006         0.644760013         0.279159993
     0.355239987         0.177619994         0.279159993
     0.489046663         0.510953307         0.612493336
     0.489046663         0.978093326         0.612493336
     0.021906653         0.510953307         0.612493336
     0.155713335         0.844286680         0.945826650
     0.155713335         0.311426669         0.945826650
     0.688573301         0.844286680         0.945826650
     0.000000000         0.000000000         0.742529988
     0.666666687         0.333333343         0.075863324
     0.333333343         0.666666687         0.409196645
     0.000000000         0.000000000         0.486999989
     0.666666687         0.333333343         0.820333302
     0.333333343         0.666666687         0.153666660
```

I–4

```
Im4
 1.00000000000000000
    5.2638481694550574    0.0000000190539741    0.0000000000000000
   -0.0000000190539741    5.2638481694830466    0.0000000000000000
    0.0000000000000000    0.0000000000000000    3.6145875030491039
  N    F
    2    10
Direct
  0.0000000000000000  0.0000000000000000  0.0000000000000000
```

```
0.5000000000000000  0.5000000000000000  0.5000000000000000
0.6906959239421302  0.4473391931147295  0.2842978500721502
0.3093040760578699  0.5526608068852704  0.2842978500721502
0.4473391931147295  0.3093040760578699  0.7157021789278486
0.5526608068852704  0.6906959239421301  0.7157021789278486
0.1906959239421301  0.9473391931147296  0.7842978210721514
0.8093040760578698  0.0526608068852704  0.7842978210721514
0.9473391931147296  0.8093040760578699  0.2157021499278498
0.0526608068852704  0.1906959239421301  0.2157021499278498
0.0000000000000000  0.5000000000000000  0.7500000000000000
0.5000000000000000  0.0000000000000000  0.2500000000000000
```

P–1

```
Pm1
 1.00000000000000000
   4.0381208629365757   0.0692241556353655  -0.3013607201912900
  -1.8388476066351371   3.7020239729576354  -0.0055006034988891
  -0.8405154990568953  -2.4497017733987123   6.8648550980811907
  N   F
   2   10
Direct
 0.0735011346841159  0.2391656007892571  0.2539431448887080
 0.9264988883158826  0.7608343852107416  0.7460568851112803
 0.3992204218878211  0.6540538832297815  0.3857377858978495
 0.6007795481121762  0.3459461167702043  0.6142621841021552
 0.3288615070224843  0.1268037596616398  0.2215944673858699
 0.6711384929775158  0.8731962323383595  0.7784055616141288
 0.9296778803568756  0.3770528997800159  0.1306314738442961
 0.0703221196431174  0.6229471002199841  0.8693684961557085
 0.0457329456511985  0.8065844507056338  0.5909116211582074
 0.9542670243487992  0.1934155492943662  0.4090883788417928
 0.2783332343032509  0.1831948917454731  0.8850067083010054
 0.7216667356967468  0.8168051082545269  0.1149932986989918
```

I4/m

```
I4_m
 1.00000000000000000
   5.9458639042836721  -0.0005085816001952   0.0000000000000000
  -0.0005022293795611   5.9439291912853118   0.0000000000000000
   0.0000000000000000   0.0000000000000000   8.3759546509852623
  N   F
   6   30
Direct
 0.0000000000000000  0.5000000000000000  0.2500388220641554
 0.0000000000000000  0.5000000000000000  0.7499611779358446
 0.5000000000000000  0.0000000000000000  0.2499611779358446
 0.5000000000000000  0.0000000000000000  0.7500388220641554
 0.0000000000000000  0.0000000000000000  0.5000000000000000
 0.5000000000000000  0.5000000000000000  0.0000000000000000
 0.0000000000000000  0.0000000000000000  0.0000000000000000
 0.5000000000000000  0.5000000000000000  0.5000000000000000
 0.6133927743749588  0.2706088173035590  0.0000000000000000
 0.3866072256250414  0.7293911676964434  0.0000000000000000
 0.7293414305993716  0.6134168324475179  0.0000000000000000
 0.2706585544006308  0.3865831675524890  0.0000000000000000
 0.1133927743749586  0.7706088323035566  0.5000000000000000
 0.8866072256250412  0.2293911676964434  0.5000000000000000
 0.2293414305993716  0.1134168324475180  0.5000000000000000
```

```
0.7706585694006284  0.8865831675524821  0.5000000000000000
0.0000000000000000  0.0000000000000000  0.6789157422573709
0.0000000000000000  0.0000000000000000  0.3210842577426362
0.5000000000000000  0.5000000000000000  0.1789157422573709
0.5000000000000000  0.5000000000000000  0.8210842577426291
0.8269662238196509  0.5522525833038432  0.3393717559291096
0.1730337761803565  0.4477474166961569  0.6606282150708848
0.1730337761803565  0.4477474166961569  0.3393717559291096
0.8269662238196509  0.5522525833038432  0.6606282150708848
0.4477780728553068  0.8269026785606808  0.3394767662691029
0.5522219271446934  0.1730973214393192  0.6605232047308914
0.5522219271446934  0.1730973214393192  0.3394767662691029
0.4477780728553068  0.8269026785606808  0.6605232047308914
0.3269662238196507  0.0522525833038502  0.8393717849291152
0.6730337761803491  0.9477474166961568  0.1606282440708905
0.6730337761803491  0.9477474166961568  0.8393717849291152
0.3269662238196507  0.0522525833038502  0.1606282440708905
0.9477780728553066  0.3269026785606808  0.8394767952691086
0.0522219271447004  0.6730973214393192  0.1605232337308972
0.0522219271447004  0.6730973214393192  0.8394767952691086
0.9477780728553066  0.3269026785606808  0.1605232337308972
```

P4/n

```
P4_n
 1.00000000000000000
     5.9981309844180943    0.0000283694390696   -0.0000001873027544
     0.0000281245792918    5.9979943314818218   -0.0000001596881872
    -0.0000000942912845    0.0000002267646359    5.5135320444675040
   N    F
     4    20
Direct
  0.7500000280959861  0.2500000488274517 -0.0000011307093163
  0.2499999719040141  0.7499999511725483  0.0000011307093164
  0.2499999968204191  0.2499999177558929  0.6041130058496887
  0.7500000031795808  0.7500000822441071  0.3958869941503044
  0.2500000188092132  0.2499999301793780  0.3339297373782249
  0.7499999811907868  0.7500000698206221  0.6660702336217692
  0.2499999370334092  0.2499999455113353  0.8887736752542775
  0.7500000629665907  0.7500000544886646  0.1112263247457296
  0.8082320429203473  0.0813241765189142  0.8660731007096020
  0.1917679570796671  0.9186758464810910  0.1339268992903981
  0.6917679614088268  0.4186759585469091  0.8660731486510889
  0.3082320385911591  0.5813240114530883  0.1339268513489111
  0.4186733456155017  0.8082347021424413  0.8660713929860842
  0.5813266243844960  0.1917652978575586  0.1339286070139159
  0.0813266037382734  0.6917651708944107  0.8660713870673805
  0.9186734192617321  0.3082348291055894  0.1339286129326195
  0.1741256643234220  0.0100486840924239  0.6075754054890523
  0.8258743356765780  0.9899512889075844  0.3924245945109547
  0.3258743247336251  0.4899511542354998  0.6075753010634297
  0.6741256752663748  0.5100488757644955  0.3924246989365776
  0.4899477376354340  0.1741199812273357  0.6075795635982052
  0.5100522923645614  0.8258800187726643  0.3924204364018020
  0.0100522474616085  0.3258798213791301  0.6075794584677594
  0.9899477255383927  0.6741201786208700  0.3924205415322479
```

VI. References

1. Grant, D. J., Wang, T.-H., Vasiliu, M., Dixon, D. a & Christe, K. O. F^+ and F^- affinities of simple N_xF_y and O_xF_y compounds. *Inorg. Chem.* **50,** 1914–25 (2011).
2. Frisch, M. J. *et al.* Gaussian 09, Revision D.01. (2013).
3. Chase, M. W. J. *NIST JANAF Thermochemical Tables*. (American Institute of Physics, 1998).
4. Clark, S. J. *et al.* First principles methods using CASTEP. *Z. Kristallogr.* **220** (2005).
